# 8-Chloro-5,5-dimethyl-5,6-dihydro­tetra­zolo[1,5-*c*]quinazoline

**DOI:** 10.1107/S160053681004688X

**Published:** 2010-11-20

**Authors:** Hoong-Kun Fun, Chin Sing Yeap, J. Gowda, A. M. A. Khader, Balakrishna Kalluraya

**Affiliations:** a X-ray Crystallography Unit, School of Physics, Universiti Sains Malaysia, 11800 USM, Penang, Malaysia; b Department of Studies in Chemistry, Mangalore University, Mangalagangotri, Mangalore 574 199, India

## Abstract

In the title compound, C_10_H_10_ClN_5_, the tetra­zole ring and the phenyl ring make a dihedral angle of 7.7 (2)°. The hexa­hydro­pyrimidine ring adopts a screw-boat conformation. In the crystal, inter­molecular bifurcated N—H⋯(N,N) hydrogen bonds link the mol­ecules into [001] chains.

## Related literature

For applications of tetra­zole derivatives, see: Upadhayaya *et al.* (2004 ▶); Poonian *et al.* (1976 ▶); Ismail *et al.* (2006 ▶); Mulwad & Kewat (2008 ▶); Uchida *et al.* (1989 ▶). For ring conformations, see: Boeyens (1978 ▶). For the stability of the temperature controller used in the data collection, see: Cosier & Glazer (1986 ▶).
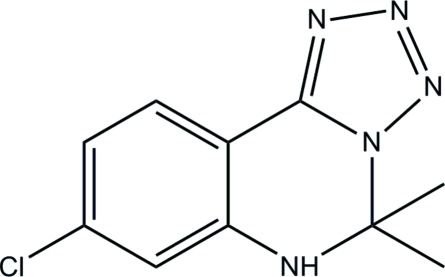


## Experimental

### 

#### Crystal data

C_10_H_10_ClN_5_
*M* *_r_* = 235.68
Monoclinic, 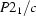

*a* = 6.8324 (16) Å
*b* = 21.532 (5) Å
*c* = 9.4337 (16) Å
β = 130.823 (11)°
*V* = 1050.2 (4) Å^3^
*Z* = 4
Mo *K*α radiation
μ = 0.34 mm^−1^
*T* = 100 K
0.16 × 0.11 × 0.05 mm

#### Data collection

Bruker APEXII DUO CCD diffractometer
Absorption correction: multi-scan (*SADABS*; Bruker, 2009 ▶) *T*_min_ = 0.948, *T*_max_ = 0.982
9660 measured reflections
2412 independent reflections
1777 reflections with *I* > 2σ(*I*)
*R*_int_ = 0.066

#### Refinement

*R*[*F* ^2^ > 2σ(*F* ^2^)] = 0.053
*wR*(*F* ^2^) = 0.137
*S* = 1.12
2412 reflections
151 parameters
H atoms treated by a mixture of independent and constrained refinement
Δρ_max_ = 0.61 e Å^−3^
Δρ_min_ = −0.31 e Å^−3^

### 

Data collection: *APEX2* (Bruker, 2009 ▶); cell refinement: *SAINT* (Bruker, 2009 ▶); data reduction: *SAINT*; program(s) used to solve structure: *SHELXTL* (Sheldrick, 2008 ▶); program(s) used to refine structure: *SHELXTL*; molecular graphics: *SHELXTL*; software used to prepare material for publication: *SHELXTL* and *PLATON* (Spek, 2009 ▶).

## Supplementary Material

Crystal structure: contains datablocks global, I. DOI: 10.1107/S160053681004688X/hb5733sup1.cif

Structure factors: contains datablocks I. DOI: 10.1107/S160053681004688X/hb5733Isup2.hkl

Additional supplementary materials:  crystallographic information; 3D view; checkCIF report

## Figures and Tables

**Table 1:** Hydrogen-bond geometry (Å, °)

*D*—H⋯*A*	*D*—H	H⋯*A*	*D*⋯*A*	*D*—H⋯*A*
N5—H1*N*5⋯N1^i^	0.85 (4)	2.35 (4)	3.190 (3)	173 (6)
N5—H1*N*5⋯N2^i^	0.85 (4)	2.57 (4)	3.326 (3)	150 (4)

Symmetry code: (i) 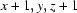.

**Fig. 1.**
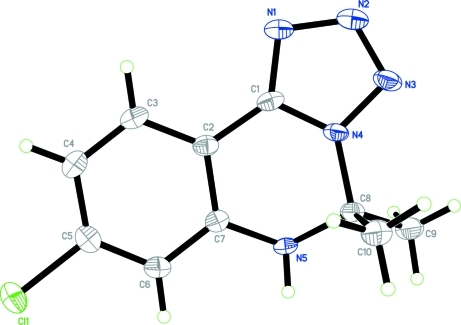
The molecular structure of the title compound with 50% probability ellipsoids for non-H atoms.

**Fig. 2.**
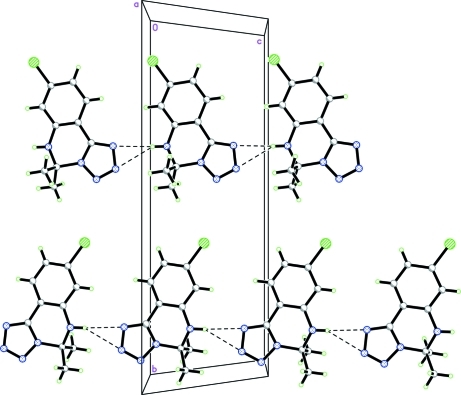
The crystal packing of title compound, viewed down b axis, showing the molecules are linked into chains along c axis. Intermolecular hydrogen bonds are shown as dashed lines.

## supplementary crystallographic information

### Comment

A number of tetrazole derivatives were reported as to be antifungal agents (Upadhayaya *et al.*, 2004), antiviral agents (Poonian *et al.*, 1976), angiotensin II AT^1^ receptor antagonists (Ismail *et al.*, 2006), antibacterial agents (Mulwad & Kewat, 2008) and anti-ulcer agents (Uchida *et al.*, 1989). On the basis of these considerations, our particular attention was directed to synthesize some tetrazole derivatives.

The title compound is a three fused-ring structure (Fig. 1). The tetrazole ring and the phenyl ring make dihedral angle of 7.7 (2)°. The hexahydropyrimidine ring adopts a screw-boat conformation, with puckering amplitude Q = 0.308 (3) Å, θ = 61.8 (6)°, φ = 270.4 (7)° (Boeyens, 1978). In the crystal structure, intermolecular bifurcated N5—H1N5···N1 and N5—H1N5···N2 hydrogen bonds link the molecules into chains along *c* axis (Fig. 2, Table 1).

### Experimental

To a solution of 2-amino-4-chlorobenzonitrile (4.2 mmol) in *N*,*N*-dimethylformamide was added ammonium chloride (3 eq) and sodium azide (3 eq). The resulting reaction mixture was refluxed for 12 h. The completion of reaction was checked by TLC (100% EA). The reaction mixture was poured into ice-water after cooling to RT and acidified to give tetrazoles as a white mass. The resulting compound was then condensed with acetone to get the title compound: colourless plates were obtained by crystallization from acetone under slow evaporation (Mp*.* 501 K).

### Refinement

The N-bound hydrogen atom was located from difference Fourier map and refined freely. The rest of hydrogen atoms were positioned geometrically [C–H = 0.93 or 0.96 Å] and refined using a riding model [*U*_iso_(H) = 1.2 or 1.5*U*_eq_]. A rotating-group model were applied for methyl groups.

### Crystal data

**Table:**

C_10_H_10_ClN_5_	*F*(000) = 488
*M**_r_* = 235.68	*D*_x_ = 1.491 Mg m^−^^3^
Monoclinic, *P*2_1_/*c*	Mo *K*α radiation, λ = 0.71073 Å
Hall symbol: -P 2ybc	Cell parameters from 1888 reflections
*a* = 6.8324 (16) Å	θ = 3.0–29.9°
*b* = 21.532 (5) Å	µ = 0.34 mm^−^^1^
*c* = 9.4337 (16) Å	*T* = 100 K
β = 130.823 (11)°	Plate, colourless
*V* = 1050.2 (4) Å^3^	0.16 × 0.11 × 0.05 mm
*Z* = 4	

### Data collection

**Table:**

Bruker APEXII DUO CCD diffractometer	2412 independent reflections
Radiation source: fine-focus sealed tube	1777 reflections with *I* > 2σ(*I*)
graphite	*R*_int_ = 0.066
φ and ω scans	θ_max_ = 27.5°, θ_min_ = 1.9°
Absorption correction: multi-scan (*SADABS*; Bruker, 2009)	*h* = −8→8
*T*_min_ = 0.948, *T*_max_ = 0.982	*k* = −27→27
9660 measured reflections	*l* = −12→12

### Refinement

**Table:**

Refinement on *F*^2^	Primary atom site location: structure-invariant direct methods
Least-squares matrix: full	Secondary atom site location: difference Fourier map
*R*[*F*^2^ > 2σ(*F*^2^)] = 0.053	Hydrogen site location: inferred from neighbouring sites
*wR*(*F*^2^) = 0.137	H atoms treated by a mixture of independent and constrained refinement
*S* = 1.12	*w* = 1/[σ^2^(*F*_o_^2^) + (0.0361*P*)^2^ + 1.9098*P*] where *P* = (*F*_o_^2^ + 2*F*_c_^2^)/3
2412 reflections	(Δ/σ)_max_ < 0.001
151 parameters	Δρ_max_ = 0.61 e Å^−^^3^
0 restraints	Δρ_min_ = −0.31 e Å^−^^3^

### Special details

**Table:**

Experimental. The crystal was placed in the cold stream of an Oxford Cryosystems Cobra open-flow nitrogen cryostat (Cosier & Glazer, 1986) operating at 100.0 (1) K.
Geometry. All e.s.d.'s (except the e.s.d. in the dihedral angle between two l.s. planes) are estimated using the full covariance matrix. The cell e.s.d.'s are taken into account individually in the estimation of e.s.d.'s in distances, angles and torsion angles; correlations between e.s.d.'s in cell parameters are only used when they are defined by crystal symmetry. An approximate (isotropic) treatment of cell e.s.d.'s is used for estimating e.s.d.'s involving l.s. planes.
Refinement. Refinement of *F*^2^ against ALL reflections. The weighted *R*-factor *wR* and goodness of fit *S* are based on *F*^2^, conventional *R*-factors *R* are based on *F*, with *F* set to zero for negative *F*^2^. The threshold expression of *F*^2^ > σ(*F*^2^) is used only for calculating *R*-factors(gt) *etc*. and is not relevant to the choice of reflections for refinement. *R*-factors based on *F*^2^ are statistically about twice as large as those based on *F*, and *R*- factors based on ALL data will be even larger.

### Fractional atomic coordinates and isotropic or equivalent isotropic displacement parameters (Å^2^)

**Table:**

	*x*	*y*	*z*	*U*_iso_*/*U*_eq_	
Cl1	0.68351 (15)	0.62515 (4)	0.46138 (11)	0.0285 (2)	
N1	0.0252 (5)	0.85800 (13)	−0.2050 (3)	0.0225 (6)	
N2	0.0079 (5)	0.92121 (14)	−0.2261 (3)	0.0276 (6)	
N3	0.1672 (5)	0.94937 (13)	−0.0661 (3)	0.0248 (6)	
N4	0.2938 (4)	0.90385 (12)	0.0634 (3)	0.0192 (5)	
N5	0.6368 (5)	0.85848 (12)	0.3484 (3)	0.0210 (6)	
C1	0.2056 (5)	0.84885 (14)	−0.0218 (4)	0.0190 (6)	
C2	0.3106 (5)	0.79248 (14)	0.0880 (4)	0.0185 (6)	
C3	0.2129 (6)	0.73316 (15)	0.0145 (4)	0.0216 (7)	
H3A	0.0704	0.7285	−0.1122	0.026*	
C4	0.3259 (6)	0.68153 (15)	0.1280 (4)	0.0234 (7)	
H4A	0.2613	0.6420	0.0797	0.028*	
C5	0.5397 (6)	0.69013 (15)	0.3174 (4)	0.0213 (6)	
C6	0.6402 (6)	0.74777 (15)	0.3947 (4)	0.0209 (6)	
H6A	0.7816	0.7518	0.5218	0.025*	
C7	0.5276 (5)	0.80012 (14)	0.2800 (4)	0.0176 (6)	
C8	0.4845 (5)	0.91539 (14)	0.2689 (4)	0.0189 (6)	
C9	0.6573 (6)	0.96910 (15)	0.3100 (4)	0.0271 (7)	
H9A	0.7358	0.9608	0.2569	0.041*	
H9B	0.7896	0.9742	0.4431	0.041*	
H9C	0.5561	1.0064	0.2561	0.041*	
C10	0.3352 (6)	0.92761 (16)	0.3348 (4)	0.0254 (7)	
H10A	0.2294	0.8923	0.3065	0.038*	
H10B	0.2275	0.9635	0.2718	0.038*	
H10C	0.4549	0.9347	0.4675	0.038*	
H1N5	0.751 (8)	0.8595 (18)	0.467 (6)	0.039 (11)*	

### Atomic displacement parameters (Å^2^)

**Table:**

	*U*^11^	*U*^22^	*U*^33^	*U*^12^	*U*^13^	*U*^23^
Cl1	0.0280 (4)	0.0248 (4)	0.0334 (4)	0.0052 (3)	0.0204 (3)	0.0085 (4)
N1	0.0187 (12)	0.0307 (16)	0.0131 (12)	0.0000 (10)	0.0082 (10)	0.0003 (11)
N2	0.0240 (13)	0.0355 (17)	0.0148 (12)	0.0021 (12)	0.0089 (11)	0.0035 (12)
N3	0.0251 (13)	0.0289 (15)	0.0147 (12)	0.0052 (11)	0.0106 (11)	0.0061 (11)
N4	0.0191 (12)	0.0225 (14)	0.0107 (11)	0.0023 (10)	0.0074 (10)	0.0021 (10)
N5	0.0173 (12)	0.0213 (14)	0.0104 (12)	−0.0010 (10)	0.0029 (10)	−0.0017 (10)
C1	0.0154 (13)	0.0267 (16)	0.0135 (13)	−0.0013 (12)	0.0088 (11)	−0.0040 (12)
C2	0.0167 (13)	0.0237 (17)	0.0151 (13)	0.0009 (12)	0.0104 (11)	−0.0009 (12)
C3	0.0182 (14)	0.0282 (18)	0.0166 (14)	−0.0047 (12)	0.0105 (12)	−0.0053 (13)
C4	0.0237 (15)	0.0227 (17)	0.0253 (16)	−0.0048 (13)	0.0166 (13)	−0.0063 (13)
C5	0.0214 (14)	0.0236 (17)	0.0257 (15)	0.0044 (12)	0.0184 (13)	0.0044 (13)
C6	0.0178 (14)	0.0259 (17)	0.0165 (14)	0.0015 (12)	0.0101 (12)	0.0001 (12)
C7	0.0162 (13)	0.0204 (16)	0.0171 (13)	−0.0006 (11)	0.0113 (11)	−0.0016 (12)
C8	0.0174 (13)	0.0207 (16)	0.0135 (13)	−0.0005 (12)	0.0079 (11)	0.0006 (12)
C9	0.0288 (16)	0.0279 (18)	0.0178 (14)	−0.0080 (14)	0.0122 (13)	−0.0009 (13)
C10	0.0240 (15)	0.0300 (19)	0.0195 (15)	0.0002 (13)	0.0130 (13)	−0.0006 (13)

### Geometric parameters (Å, °)

**Table:**

Cl1—C5	1.739 (3)	C3—H3A	0.9300
N1—C1	1.326 (4)	C4—C5	1.396 (4)
N1—N2	1.369 (4)	C4—H4A	0.9300
N2—N3	1.297 (4)	C5—C6	1.374 (4)
N3—N4	1.349 (3)	C6—C7	1.394 (4)
N4—C1	1.334 (4)	C6—H6A	0.9300
N4—C8	1.489 (3)	C8—C9	1.511 (4)
N5—C7	1.386 (4)	C8—C10	1.525 (4)
N5—C8	1.458 (4)	C9—H9A	0.9600
N5—H1N5	0.85 (4)	C9—H9B	0.9600
C1—C2	1.445 (4)	C9—H9C	0.9600
C2—C3	1.397 (4)	C10—H10A	0.9600
C2—C7	1.413 (4)	C10—H10B	0.9600
C3—C4	1.378 (4)	C10—H10C	0.9600
			
C1—N1—N2	104.8 (2)	C5—C6—C7	119.2 (3)
N3—N2—N1	111.6 (2)	C5—C6—H6A	120.4
N2—N3—N4	105.5 (3)	C7—C6—H6A	120.4
C1—N4—N3	109.3 (2)	N5—C7—C6	121.0 (3)
C1—N4—C8	126.8 (2)	N5—C7—C2	119.8 (3)
N3—N4—C8	123.7 (2)	C6—C7—C2	119.0 (3)
C7—N5—C8	122.4 (2)	N5—C8—N4	104.6 (2)
C7—N5—H1N5	113 (3)	N5—C8—C9	109.6 (2)
C8—N5—H1N5	113 (3)	N4—C8—C9	109.5 (2)
N1—C1—N4	108.8 (3)	N5—C8—C10	112.2 (2)
N1—C1—C2	131.4 (3)	N4—C8—C10	108.1 (2)
N4—C1—C2	119.8 (2)	C9—C8—C10	112.4 (3)
C3—C2—C7	120.2 (3)	C8—C9—H9A	109.5
C3—C2—C1	124.0 (3)	C8—C9—H9B	109.5
C7—C2—C1	115.7 (3)	H9A—C9—H9B	109.5
C4—C3—C2	120.6 (3)	C8—C9—H9C	109.5
C4—C3—H3A	119.7	H9A—C9—H9C	109.5
C2—C3—H3A	119.7	H9B—C9—H9C	109.5
C3—C4—C5	118.3 (3)	C8—C10—H10A	109.5
C3—C4—H4A	120.9	C8—C10—H10B	109.5
C5—C4—H4A	120.9	H10A—C10—H10B	109.5
C6—C5—C4	122.7 (3)	C8—C10—H10C	109.5
C6—C5—Cl1	118.8 (2)	H10A—C10—H10C	109.5
C4—C5—Cl1	118.5 (2)	H10B—C10—H10C	109.5
			
C1—N1—N2—N3	−0.1 (3)	C4—C5—C6—C7	−0.8 (4)
N1—N2—N3—N4	0.4 (3)	Cl1—C5—C6—C7	179.5 (2)
N2—N3—N4—C1	−0.7 (3)	C8—N5—C7—C6	−153.7 (3)
N2—N3—N4—C8	−175.8 (2)	C8—N5—C7—C2	31.3 (4)
N2—N1—C1—N4	−0.3 (3)	C5—C6—C7—N5	−174.1 (3)
N2—N1—C1—C2	179.9 (3)	C5—C6—C7—C2	1.0 (4)
N3—N4—C1—N1	0.6 (3)	C3—C2—C7—N5	174.4 (3)
C8—N4—C1—N1	175.6 (2)	C1—C2—C7—N5	−4.6 (4)
N3—N4—C1—C2	−179.6 (2)	C3—C2—C7—C6	−0.7 (4)
C8—N4—C1—C2	−4.6 (4)	C1—C2—C7—C6	−179.7 (2)
N1—C1—C2—C3	−7.2 (5)	C7—N5—C8—N4	−38.5 (3)
N4—C1—C2—C3	173.0 (3)	C7—N5—C8—C9	−155.9 (3)
N1—C1—C2—C7	171.7 (3)	C7—N5—C8—C10	78.4 (3)
N4—C1—C2—C7	−8.0 (4)	C1—N4—C8—N5	25.7 (4)
C7—C2—C3—C4	0.2 (4)	N3—N4—C8—N5	−159.9 (2)
C1—C2—C3—C4	179.2 (3)	C1—N4—C8—C9	143.2 (3)
C2—C3—C4—C5	−0.1 (4)	N3—N4—C8—C9	−42.5 (4)
C3—C4—C5—C6	0.4 (4)	C1—N4—C8—C10	−94.0 (3)
C3—C4—C5—Cl1	−179.9 (2)	N3—N4—C8—C10	80.3 (3)

### Hydrogen-bond geometry (Å, °)

**Table:**

*D*—H···*A*	*D*—H	H···*A*	*D*···*A*	*D*—H···*A*
N5—H1N5···N1^i^	0.85 (4)	2.35 (4)	3.190 (3)	173 (6)
N5—H1N5···N2^i^	0.85 (4)	2.57 (4)	3.326 (3)	150 (4)

Symmetry codes: (i) *x*+1, *y*, *z*+1.
